# Impact of spiking neurons leakages and network recurrences on event-based spatio-temporal pattern recognition

**DOI:** 10.3389/fnins.2023.1244675

**Published:** 2023-11-24

**Authors:** Mohamed Sadek Bouanane, Dalila Cherifi, Elisabetta Chicca, Lyes Khacef

**Affiliations:** ^1^Institute of Electrical and Electronic Engineering, University of Boumerdes, Boumerdes, Algeria; ^2^Bio-Inspired Circuits and Systems Lab, Zernike Institute for Advanced Materials, University of Groningen, Groningen, Netherlands; ^3^Groningen Cognitive Systems and Materials Center, University of Groningen, Groningen, Netherlands

**Keywords:** event-based sensors, digital neuromorphic architectures, spiking neural networks, spatio-temporal patterns, neurons leakages, neural heterogeneity, network recurrences

## Abstract

Spiking neural networks coupled with neuromorphic hardware and event-based sensors are getting increased interest for low-latency and low-power inference at the edge. However, multiple spiking neuron models have been proposed in the literature with different levels of biological plausibility and different computational features and complexities. Consequently, there is a need to define the right level of abstraction from biology in order to get the best performance in accurate, efficient and fast inference in neuromorphic hardware. In this context, we explore the impact of synaptic and membrane leakages in spiking neurons. We confront three neural models with different computational complexities using feedforward and recurrent topologies for event-based visual and auditory pattern recognition. Our results showed that, in terms of accuracy, leakages are important when there are both temporal information in the data and explicit recurrence in the network. Additionally, leakages do not necessarily increase the sparsity of spikes flowing in the network. We also investigated the impact of heterogeneity in the time constant of leakages. The results showed a slight improvement in accuracy when using data with a rich temporal structure, thereby validating similar findings obtained in previous studies. These results advance our understanding of the computational role of the neural leakages and network recurrences, and provide valuable insights for the design of compact and energy-efficient neuromorphic hardware for embedded systems.

## 1 Introduction

Over the last decade, Artificial Neural Networks (ANNs) have been increasingly attracting interest in both academia and industry as a consequence of the explosion of open data and the high computing power of today's computers for training and inference. The state-of-the-art performance of deep neural networks on various pattern recognition tasks has given neural networks a leading role in Machine Learning (ML) algorithms and Artificial Intelligence (AI) research. However, the technological drive that has supported Moore's Law for 50 years and the increasing computing power of conventional processors is reaching a physical limit and is predicted to flatten by 2025 (Shalf, 2020). Hence, deep learning progress with current models and implementations will become technically, economically, and environmentally unsustainable (Thompson et al., 2020, 2021). This limit is particularly prohibitive when targeting edge applications in embedded systems with severe constraints in latency and energy consumption (Rabaey et al., 2019).

Neuromorphic computing is a promising solution that takes inspiration from the biological brain which can reliably learn and process complex cognitive tasks at a very low power consumption. On the one hand, neuromorphic sensors are event-based sensors and capture information with a high temporal resolution and high spatio-temporal sparsity at low-latency and low-power consumption (Liu et al., 2010; Gallego et al., 2022). On the other hand, neuromorphic processors are asynchronous and use parallel and distributed implementations of synapses and neurons where memory and computation are co-localized (Mead and Conway, 1980; Chicca et al., 2014), hence adapting the hardware to the computation model (Schuman et al., 2017; Bouvier et al., 2019). Spiking Neural Networks (SNNs) are the third generation of artificial neural models (Maass, 1997) that are investigated to exploit the advantages of event-based sensing and asynchronous processing at the algorithmic level.

Inspired from the neuroscience literature, Spiking Neural Networks (SNNs) show promising performance in embedded spatio-temporal pattern recognition (Davies et al., 2021). For example, compared to a conventional approach using formal neural networks on an embedded Nvidia Jetson GPU, SNNs on the Intel Loihi neuromorphic chip (Davies et al., 2018) achieve a gain in energy-efficiency of 30× in multimodal (vision and EMG) hand gesture recognition (Ceolini et al., 2020) and 500× in tactile braille letters recognition (Muller-Cleve et al., 2022), at the cost of a loss in accuracy depending on the application. Multiple models of spiking neurons have been proposed in the literature (Hodgkin and Huxley, 1952; Kistler et al., 1997; Izhikevich, 2003) and implemented in hardware (Indiveri et al., 2011) with different levels of biological plausibility and computational complexity. However, there is a lack of understanding of how each of the factors determining the biological neuronal response can be effectively used in learning and inference. A key question for advancing the field is therefore to identify the right level of abstraction inspired from biology to achieve the best inference performance within strict constrains in speed/latency and power efficiency on neuromorphic hardware.

This work attempts to partially answer this question by studying the effect of spiking neurons leakages in feedforward and recurrent neural networks for event-based visual and auditory pattern recognition tasks, in terms of accuracy and spiking activity. Today, digital neuromorphic chips from academia and industry use both non-leaky [e.g., SPLEAT (Abderrahmane et al., 2022) and DynapCNN (Liu et al., 2019)] and leaky [e.g., MorphIC (Frenkel et al., 2019) and Loihi (Davies et al., 2018)] spiking neurons. Understanding the computational role of the leakages provides insights for the hardware architecture of neuromorphic processors as they require extra circuitry overheads (Khacef et al., 2018). In Section 2, we introduce the spiking neuron models and present the training methodology. In Section 3, we present our grid search experiments and a detailed analysis of the resulting performance trends across different spiking neuron models, leakage parameters, network topologies, as well as time constant heterogeneity. Finally, in Sections 4 and 5 we discuss the results, highlighting the main insights, limits and outlook of our work.

## 2 Methods

In this section, we introduce the used network typologies along with their spiking neuron model that are characterized with different levels of biological abstraction. We also introduce the surrogate gradient decent approach used in this work to overcome the all-or-nothing behavior of the binary spiking non-linearity.

### 2.1 Spiking neural network topologies

**Figure 1:**
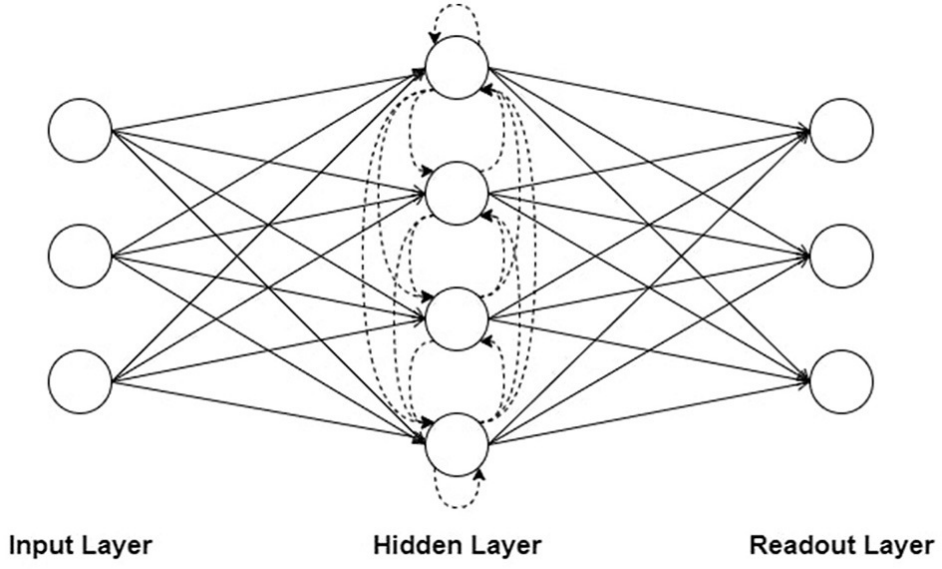
Simplified diagram representation of: FSNN (without dashed lines) vs. RSNN (including dashed lines) network topologies.

In this work, we adopted a necessary and sufficient minimal architecture widely used as a universal approximator (Maass et al., 2002; Neftci et al., 2019) consisting of three layers of spiking neurons: an input layer, a hidden layer with or without recurrent connections which contains *N* = 200 neuron, and a readout layer with infinite threshold (non-spiking neurons) where the membranes potentials are used to generate predictions. In the case of a hidden layer with recurrent connections, the used recurrent network is a Hopfield-like neural network where neurons are fully interconnected. Throughout the paper, we refer to Feed-forward SNNs as FSNNs and Recurrently connected SNNs as RSNNs. Figure 1 demonstrate a simplified diagrammatic representation of the networks.

### 2.2 Spiking neuron models

The standard spiking neuron model is formally described as a time continuous dynamical system with the differential equation (Gerstner et al., 2014):


(1) $$\begin{array}{l} \tau_{mem}\frac{\text{d}U_{i}^{\left(l\right)}\left(t\right)}{\text{d}t}=-\left(U_{i}^{\left(l\right)}\left(t\right)-U_{rest}\right)+RI_{i}^{\left(l\right)}\left(t\right) \end{array}$$

where *U*_*i*_(*t*) is the membrane potential that characterizes the hidden state of the neuron, *U*_*rest*_ is the resting potential, τ_*mem*_ is the membrane time constant, *R* is the input resistance, and *I*_*i*_(*t*) is the input current. The hidden state of each neuron, however, is not directly communicated to downstream neurons. When the membrane potential *U*_*i*_ reaches the firing threshold ϑ, the neuron fires an action potential (or a “spike”) and the membrane potential *U*_*i*_ is reset to its resting potential *U*_*rest*_. If we consider spikes to be point processes for which their spike width is zero in the limit, then a spike train $S_{j}^{\left(l\right)}\left(t\right)$ is denoted with the sum of Dirac delta functions $S_{j}^{\left(l\right)}\left(t\right)=\underset{s\in C_{j}^{\left(l\right)}}{\sum }\delta \left(t-s\right)$ such that *s* iterate over the firing times $C_{j}^{\left(l\right)}$ of neuron *j* from layer *l*. Spikes are communicated to downstream neurons and trigger postsynaptic currents. A common first-order approximation to model the temporal dynamics of postsynaptic currents are exponentially decaying currents that sum linearly:


(2) $$\begin{array}{l} \frac{\text{d}I_{i}{\left(t\right)}^{\left(l\right)}}{\text{d}t}=-\frac{I_{i}^{\left(l\right)}\left(t\right)}{\tau_{syn}}+\underset{j}{\sum }W_{ij}^{\left(l\right)}S_{j}^{\left(l-1\right)}\left(t\right)+\underset{j}{\sum }V_{ij}^{\left(l\right)}S_{j}^{\left(l\right)}\left(t\right) \end{array}$$

where we have introduced the synaptic decay time constant τ_*syn*_, and the synaptic weight matrices: $W_{ij}^{\left(l\right)}$ for feed-forward connections, and $V_{ij}^{\left(l\right)}$ for explicit recurrent connections within each layer.

It is customary to approximate the solutions to the above equations in discrete time assuming a small simulation time step Δ*t* > 0. With no loss of generality, we assume *U*_*rest*_ = 0, *R* = 1, and the firing threshold ϑ = 1. The output spike train $S_{i}^{\left(l\right)}\left[t\right]$ of neuron *i* in layer *l* is expressed as $S_{i}^{\left(l\right)}\left[t\right]\equiv \Theta \left(U_{i}^{\left(l\right)}\left[t\right]-\vartheta \right)$ where Θ is the Heaviside step function such that $S_{i}^{\left(l\right)}\left[t\right]\in \left\{0,1\right\}$. *t* is used to denote the time step to indicate discrete time. The synaptic and membrane dynamics expressed, respectively, by Equation (2) and Equation (1) become (Neftci et al., 2019):


(3) $$\begin{array}{l} I_{i}^{\left(l\right)}\left[t\right]=\alpha I_{i}^{\left(l\right)}\left[t-1\right]+\underset{j}{\sum }W_{ij}^{\left(l\right)}S_{j}^{\left(l-1\right)}\left[t-1\right]+\underset{j}{\sum }V_{ij}^{\left(l\right)}S_{j}^{\left(l\right)}\left[t-1\right] \end{array}$$

(4) $$\begin{array}{l} U_{i}^{\left(l\right)}\left[t\right]=\left(\beta U_{i}^{\left(l\right)}\left[t-1\right]+I_{i}^{\left(l\right)}\left[t\right]\right)\times \left(1-S_{i}^{\left(l\right)}\left[t-1\right]\right) \end{array}$$

where the decay strengths are given by $\alpha \equiv e^{-\frac{\Delta t}{\tau_{syn}}}$ and $\beta \equiv e^{-\frac{\Delta t}{\tau_{mem}}}$, such that 0 < α < 1 and 0 < β < 1 for finite and positive τ_*syn*_ and τ_*mem*_.

There exists many extensions and variations of spiking neurons models. In order to find the right level of abstraction from biology and get the best performance in accurate, efficient and fast inference, we will derive and confront three variations with variable degrees of biological plausibility: the Current-Based Leaky Integrate-and-Fire (CUBA-LIF), the Leaky Integrate-and-Fire (LIF), and the Integrate-and-Fire (IF).

#### 2.2.1 Current-based Leaky Integrate-and-Fire (CUBA-LIF)

**Figure 2:**
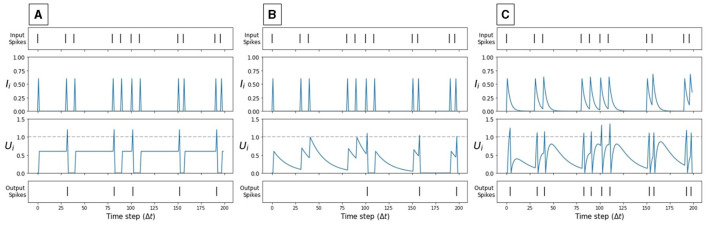
Synaptic current and membrane potential dynamics of each spiking neuron model in response to the same input spikes. **(A)** IF, **(B)** LIF, **(C)** CUBA-LIF. Only when the membrane potential reaches the neuronal firing threshold (dashed line), output spikes are generated.

The CUBA-LIF neuron is the most biologically plausible model among the three models considered in this work. It accounts for the temporal dynamics of the postsynaptic current. This neuron model is governed by Equations (5) and (6). It has two exponentially decaying terms: α*I*_*i*_ and β*U*_*i*_. The degree of the exponential decay of *I*_*i*_ and *U*_*i*_ is determined by the synaptic time constant τ_*syn*_ and membrane time constant τ_*mem*_, respectively. Figure 2C illustrate the dynamics of a CUBA-LIF neuron for some random input stimuli.


(5) $$\begin{array}{l} I_{i}^{\left(l\right)}\left[t\right]=\alpha I_{i}^{\left(l\right)}\left[t-1\right]+\underset{j}{\sum }W_{ij}^{\left(l\right)}S_{j}^{\left(l-1\right)}\left[t-1\right]+\underset{j}{\sum }V_{ij}^{\left(l\right)}S_{j}^{\left(l\right)}\left[t-1\right] \end{array}$$

(6) $$\begin{array}{l} U_{i}^{\left(l\right)}\left[t\right]=\left(\beta U_{i}^{\left(l\right)}\left[t-1\right]+I_{i}^{\left(l\right)}\left[t\right]\right)\times \left(1-S_{i}^{\left(l\right)}\left[t-1\right]\right) \end{array}$$

#### 2.2.2 Leaky Integrate-and-Fire (LIF)

The LIF neuron model is a simplification of the CUBA-LIF and it is widely used in computational neuroscience to emulate the dynamics of biological neurons (Izhikevich, 2004). It integrates the input over time with a leakage such that the internal state represented by the membrane potential goes down exponentially. As shown in Figure 2B, subsequent input spikes must be maintained for the state not to go to zero. In discrete time, the dynamics of the LIF neuron are governed by Equations (7) and (8):


(7) $$\begin{array}{l} I_{i}^{\left(l\right)}\left[t\right]=\underset{j}{\sum }W_{ij}^{\left(l\right)}S_{j}^{\left(l-1\right)}\left[t-1\right]+\underset{j}{\sum }V_{ij}^{\left(l\right)}S_{j}^{\left(l\right)}\left[t-1\right] \end{array}$$

(8) $$\begin{array}{l} U_{i}^{\left(l\right)}\left[t\right]=\left(\beta U_{i}^{\left(l\right)}\left[t-1\right]+I_{i}^{\left(l\right)}\left[t\right]\right)\times \left(1-S_{i}^{\left(l\right)}\left[t-1\right]\right) \end{array}$$

#### 2.2.3 Integrate-and-Fire (IF)

The IF neuron is a further simplification and the least biologically plausible model considered in this work. The IF model can be concisely described as a LIF neuron with no leak. It behaves as a standard integrator that keeps a running sum of its input. Thus, the internal state of the neuron is the mathematical integral of the input (Eliasmith, 2013). IF neurons do not have any inherent temporal dynamics. In discrete time, IF dynamics are governed by Equations (9) and (10):


(9) $$\begin{array}{l} I_{i}^{\left(l\right)}\left[t\right]=\underset{j}{\sum }W_{ij}^{\left(l\right)}S_{j}^{\left(l-1\right)}\left[t-1\right]+\underset{j}{\sum }V_{ij}^{\left(l\right)}S_{j}^{\left(l\right)}\left[t-1\right] \end{array}$$

(10) $$\begin{array}{l} U_{i}^{\left(l\right)}\left[t\right]=\left(U_{i}^{\left(l\right)}\left[t-1\right]+I_{i}^{\left(l\right)}\left[t\right]\right)\times \left(1-S_{i}^{\left(l\right)}\left[t-1\right]\right) \end{array}$$

Equations (9) and (10) do not have the decay (i.e., leak) parameters α and β. The parameter α is set to *zero* which causes the synapse to have an infinite leak. The current pulse width is short, it effectively looks like a weighted spike. β on the other hand is set to *one*, which causes the membrane potential to remain constant between two consecutive spikes. Figure 2A illustrates the dynamics of an IF neuron for some random input stimuli.

### 2.3 Supervised learning in SNNs

The choice of the three spiking neurons models considered is motivated by the intention of mapping existing machine learning methods to train SNNs. The aim of learning is to minimize a loss function L over the entire dataset. The gradient-based method, namely Backpropagation Trought Time (BPTT) (Goodfellow et al., 2016) was used. Before BPTT can be applied to SNNs, however, a serious challenge regarding the non-differentiability of the spiking non-linearity needs to be overcome.

BPTT requires the calculation of the derivative of the neural activation function. For a spiking neuron, however, the derivative of *S*[*t*] = Θ(*U*[*t*]−ϑ) is zero everywhere except at *U* = ϑ, where it tends to infinity as shown in Equation (11). This means the gradient will almost always be zero and no learning can take place. This behavior of the binary spiking non-linearity makes SNNs unsuitable for gradient based optimization and it is known as the “*dead neuron problem*”.


(11) $$\begin{array}{l} \frac{\partial L}{\partial W}=\frac{\partial L}{\partial S}\underset{\text{\{0,}\infty \text{\}}}{\underset{︸}{\frac{\partial S}{\partial U}}}\frac{\partial U}{\partial I}\frac{\partial I}{\partial W} \end{array}$$

In this work, we used a surrogate gradient approach (Neftci et al., 2019) to provide a continuous relaxation to the real gradients. In other words, we keep the Heaviside step function the way it is during the forward pass and change the derivative term ∂*S*/∂*U* with something that does not stop learning during the backward pass. Specifically, we selected the fast sigmoid function $\tilde{S}$ to smooth out the gradient of the Heaviside function:


(12) $$\begin{array}{l} \tilde{S}=\sigma \left(U_{i}^{\left(l\right)}\right)=\frac{U_{i}^{\left(l\right)}}{1+\tilde{\beta }|U_{i}^{\left(l\right)}|} \end{array}$$

where $\tilde{\beta }$ is the steepness parameter that modulates how smooth the surrogate function is.

In this work, cross entropy *max-over-time* loss function (Cramer et al., 2022) is chosen. When called, the maximum membrane potential value for each output neuron in the readout layer is sampled and passed through the loss function. This cross entropy loss encourages the maximum membrane potential of the correct class to increase, and suppresses the maximum membrane potential of incorrect classes. On data with batch size of *N*_*batch*_ and *N*_*class*_ output classes, {(*x*_*s*_, *y*_*s*_)∣*s* = 1, ..., *N*_*batch*_; *y*_*s*_∈{1, ..., *N*_*class*_}} the loss function takes the form:


(13) $$\begin{array}{l} L=-\frac{1}{N_{batch}}\overset{N_{batch}}{\underset{s=1}{\sum }}1\left(i=y_{s}\right)\log\left\{\frac{\exp\left(U_{i}^{\left(L\right)}\left[\tilde{t_{i}}\right]\right)}{\sum_{i=0}^{N_{class}}\exp\left(U_{i}^{\left(L\right)}\left[\tilde{t_{i}}\right]\right)}\right\} \end{array}$$

where *1* is the indicator function, and $\tilde{t}$ is the time step with the maximum membrane potential for each readout unit in the readout layer *L*, such that $\tilde{t_{i}}={\mathrm{argmax}}_{t}U_{i}^{\left(L\right)}\left[t\right]$.

The cross entropy in Equation 13 is minimized using the Adamax optimizer (Kingma and Ba, 2014).

## 3 Experiments and results

This section present all the experiments, we conducted in order to understand the effect of spiking neurons leakages and network recurrences for spike-based spatio-temporal pattern recognition and gives a detailed analysis of the results we obtained.

### 3.1 Experimental setup

We investigated the role of neurons leakages, network recurrences and neural heterogeneity by training SNNs to classify visual and auditory stimuli with varying degrees of temporal structure. Two training approaches were applied: standard training only modifies the synaptic weights, while heterogeneous training affects both the synaptic weights and the time constants.

We used two datasets. The Spiking Heidelberg Digits (SHD) (Cramer et al., 2022) is auditory and has a rich temporal structure. It is the spiking version of the Heidelberg Digits audio dataset consisting of 20 classes of spoken digits recordings from zero to nine in both English and German. It contains 8,156 training and 2,264 testing samples as shown in Table 1. Spikes in 700 input channels were generated using an artificial cochlea, where the spike timing of the input neurons is necessary to recognize each pattern (Perez-Nieves et al., 2021). SHD spike trains have a maximum duration of 1.4s and binned into 14ms bins. By contrast, the Neuromorphic MNIST (N-MNIST) (Orchard et al., 2015; Iyer et al., 2021) contains mostly spatial information. It features visual stimuli and has minimal temporal structure, as its samples are generated from static images of the frame-based MNIST benchmark by moving a neuromorphic vision sensor over each original MNIST sample resulting in 34 × 34 image with ON and OFF polarities. Therefore, the spike rate of the input neurons has sufficient information about the pattern, while the temporal component is strictly related to the movements of the vision sensor. The dataset contains 60,000 training and 10,000 testing samples as shown in Table 1 and each sample is a spike train of 360ms. In order to have the same temporal precision between the visual and the auditory datasets to allow for fair comparison on the temporal structure, N-MNIST events are also binned into 14ms bins.

**Table 1:** Hyperparameters used in our experiments.

	**N-MNIST**	**SHD**
Train/test split	60,000/10,000	8,156/2,264
Network architecture*	2312-200-10	700-200-20
Learning rate (η)	5 × 10^−3^	2 × 10^−4^
Time step (Δ*t*)	14 ms	14 ms
Steepness parameter ($\tilde{\beta }$)	100	100
Batch size	256	128
Epochs	50	200

^*^Input-hidden-readout layers.

These two datasets are chosen for this work based on their temporal structures. Expanding the evaluation to include additional datasets, such as the IBM DVS gesture dataset (Amir et al., 2017), would necessitate the use of a convolutional topology, which increases complexity beyond the scope of this work. Additionally, the conversion of an image dataset with static patterns to rate coding for SNNs is less efficient compared to ANNs due to the need to introduce an artificial temporal dimension for spike-based processing.

To allow for fair baselines comparison of performance in accuracy with previous works, we used the same train/test split suggested by the corresponding dataset authors in all of our experiments. The network architectures and common hyper-parameters in our experiments such as the batch size, number of epochs, learning rate η, and steepness parameter $\tilde{\beta }$ were tuned according to state-of-the-art results obtained from the literature (Chowdhury et al., 2021; Perez-Nieves et al., 2021; Cramer et al., 2022), as well as our own preliminary experiments. Table 1 gives a summary of all parameters used for our experiments. The performance of each configuration is quantified in terms of testing accuracy and sparsity as an estimation for dynamic energy-efficiency in neuromorphic hardware. We note that all reported error measures in this work correspond to the standard deviation of three experiments with different random initialization for the trained parameters.

### 3.2 Impact of the neural leakages

To assess the effects of the membrane and synaptic leakages, we started by confronting the three concerned neuron models: CUBA-LIF, LIF, and IF in a Feed-forward SNN (FSNN) using both datasets. Only synaptic weights are learned and leakage parameters are treated as hyper-parameters and chosen to be homogeneous (i.e., the same for all neurons). Leakage parameters α and β are tuned using the synaptic time constant τ_*syn*_ and the membrane time constant τ_*mem*_, respectively, as described by Equations (3) and (4) in the previous chapter.

#### 3.2.1 Accuracy analysis in FSNN

We started by the CUBA-LIF neuron where both leakages are of concern. This model can have a wide range of τ_*syn*_ and τ_*mem*_. We performed a grid search across a number of time constants by fixing one and changing the other. Grid search is a simple hyper-parameters tuning technique that helped us evaluate the model for a wide range of combinations to get a good understanding of the slope of change in accuracy.

Table 2A shows the SHD testing accuracy results for the chosen different combinations of τ_*mem*_ and τ_*syn*_. We can see from the time constants sweeps that τ_*mem*_ values below 420*ms* result is a significant decrease in accuracy. It is also clear that the best results seem to push τ_*syn*_, and hence α, close to zero with τ_*mem*_≥420*ms*. In other words, CUBA-LIF performs better when its dynamics are close to those of the LIF neuron. This trend is also observed for the N-MNIST as shown in Table 2B. However, we can see a 31.55% drop between the best accuracy that reached 76.94 ± 1.13% and the 45.39 ± 1.64% worst accuracy for SHD, while only a 1.59% difference between the 97.41 ± 0.07% best and the 95.82 ± 0.11% worst accuracy for N-MNIST. This suggests that the leakages seem to have greater impact on data with rich temporal structure, than on data that is intrinsically spatial and low in temporal structure.

**Table 2:** Three neuron models accuracy in FSNN.

	**LIF**	**CUBA-LIF**			
**(ms)**	τ_*syn*_ = 0 (α≈0) **(%)**	τ_*syn*_ = 14 (α≈0.368) **(%)**	τ_*syn*_ = 28 (α≈0.606) **(%)**	τ_*syn*_ = 70 (α≈0.818) **(%)**	τ_*syn*_ = 140 (α≈0.905) **(%)**
**A. SHD**					
τ_*mem*_ = 14 (β≈0.368)	38.24	45.39	49.39	56.14	60.19
τ_*mem*_ = 70 (β≈0.818)	52.88	53.60	60.15	61.63	64.24
τ_*mem*_ = 140 (β≈0.905)	65.75	66.77	67.68	67.43	65.65
τ_*mem*_ = 420 (β≈0.967)	75.06	74.51	73.58	71.02	67.63
τ_*mem*_ = 700 (β≈0.980)	77.20	75.79	73.79	71.19	66.76
τ_*mem*_ = 1120 (β≈0.987)	76.88	75.56	74.26	72.35	67.00
τ_*mem*_ = 1680 (β≈0.992)	76.50	76.94	75.99	72.61	67.54
τ_*mem*_ = ∞(β≈1)	78.36*				
**B. N-MNIST**					
τ_*mem*_ = 14 (β≈0.368)	96.00	97.14	97.27	96.98	96.92
τ_*mem*_ = 70 (β≈0.818)	97.10	97.21	97.19	96.76	96.48
τ_*mem*_ = 140 (β≈0.905)	97.45	97.39	97.03	96.67	96.33
τ_*mem*_ = 420 (β≈0.967)	97.63	97.37	96.90	96.25	95.89
τ_*mem*_ = 700 (β≈0.980)	97.48	97.41	96.95	96.28	96.08
τ_*mem*_ = 1120 (β≈0.987)	97.48	97.37	96.94	96.52	95.91
τ_*mem*_ = 1680 (β≈0.992)	97.64	97.36	96.96	96.30	95.82
τ_*mem*_ = ∞(β≈1)	97.50*				

^*^IF Neuron.

The LIF neuron has an infinite synaptic leak with τ_*syn*_ = 0 and a tunable membrane leak. So we varied τ_*mem*_ as a hyperparameter across a range of values. The results of our experiments presented in Table 2A show that the LIF neuron achieved higher accuracy than its CUBA-LIF counterpart for both datasets. but it also resulted in the lowest accuracies for very small values of τ_*mem*_.

For the IF case, there is an infinite synaptic leak similar to that of the LIF and no membrane leak. so the only possible values for time constants are τ_*mem*_ = ∞ and τ_*syn*_ = 0 which corresponds to β = 1 and α = 0, respectively. In spite of its simplicity and lack of temporal dynamics, IF neuron was able to match or even outperform the other models by reaching a testing accuracy of 78.36 ± 0.87% for SHD and 97.50 ± 0.06% for N-MNIST as shown in Table 2. This result suggests that introducing inherent temporal dynamics and increasing neuronal complexity does not necessarily lead to an improved classification accuracy even for data with rich temporal structure when using a feed-forward network.

#### 3.2.2 Sparsity analysis in FSNN

While learning performance is pivotal, it is also crucial to take into considerations the associated computational cost and energy consumption of using each model which are directly linked to the spiking activity of neurons when using a neuromorphic hardware like Intel Loihi (Davies et al., 2018) that computes asynchronously and exploits the sparsity of event-based sensing. To infer an output class, SNNs feed the input spikes over a number of time steps and perform event-based synaptic operations only when spike-inputs arrive. These synaptic operations are considered as a metric for benchmarking neuromorphic hardware (Merolla et al., 2014; Davies et al., 2018). We explored the impact of the leakages on the sparsity of each model by inferring the test set.

**Figure 3:**
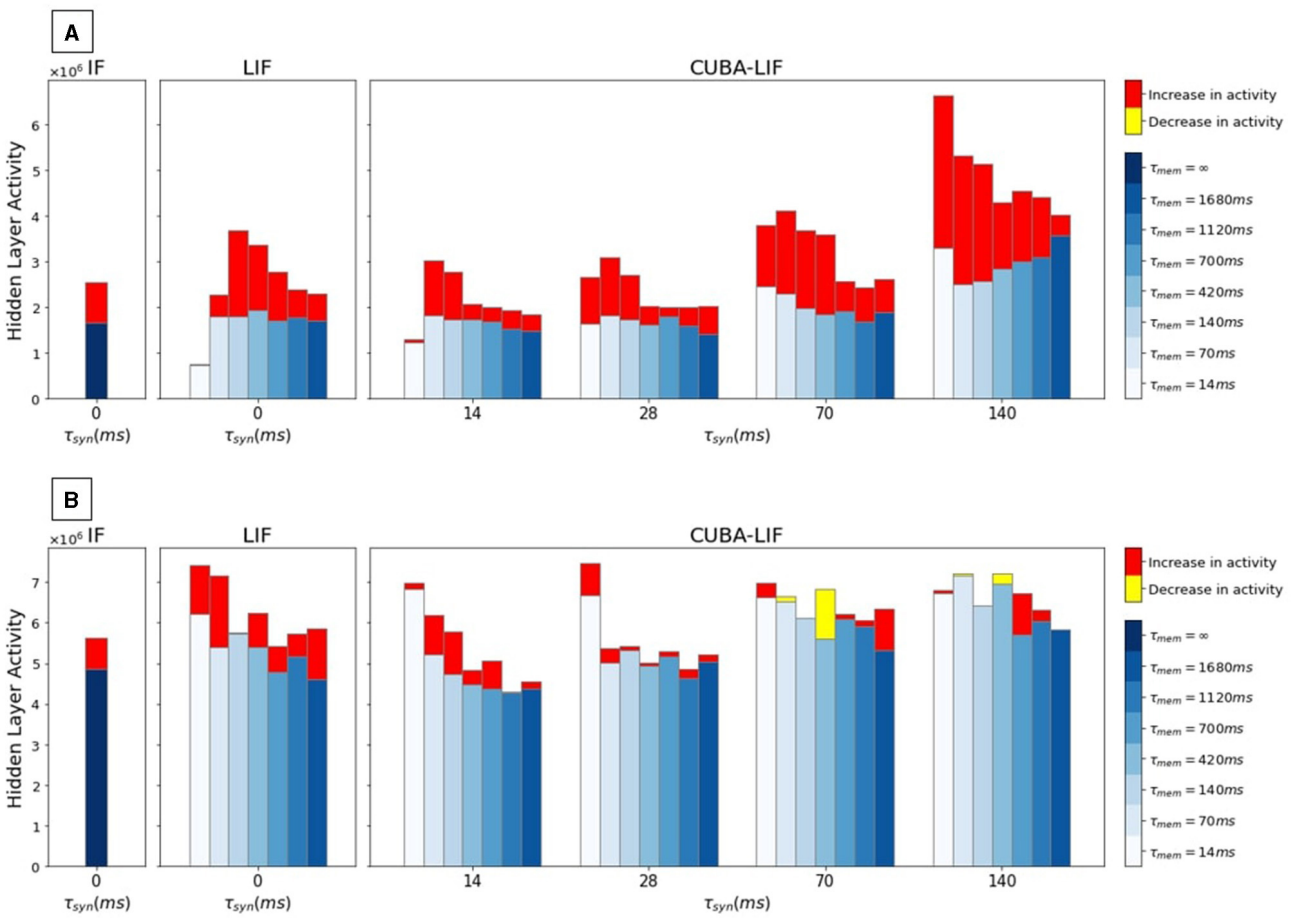
Hidden layer spiking activity with the increase/decrease caused by adding explicit recurrences for each time constants combination of three models. Each grouped set of bars corresponds to one τ_*syn*_ value while each bar within the group corresponds to one τ_*mem*_ value. **(A)** SHD, **(B)** N-MNIST.

SHD spiking activity recordings in the hidden layer plotted in Figure 3 show that the time constants combinations that led to the sparsest activity (τ_*mem*_ = 14*ms* for LIF and τ_*mem*_ = τ_*syn*_ = 14*ms* for CUBA-LIF) also resulted in the worst accuracies for both LIF and CUBA-LIF neurons. This is due to the fast decays in both membrane potential and synaptic current that result in not having enough spikes to hold the information. The same trend however cannot be observed for the N-MNIST. This could be due to the fact that leakage parameters do not have a significant impact on spatial information. Nevertheless, we can see an increase in spiking activity with higher values of τ_*syn*_ for the CUBA-LIF neuron on both datasets. Although it is more apparent on the SHD. This increase in spiking activity that resulted in a decrease in accuracy, is associated with CUBA-LIF neurons' ability to sustain input spikes over longer durations. However, more spikes do not necessarily lead to better performance, at least for our datasets.

Overall, we can observe from these results that spiking activity and accuracy are directly linked. Time constant combinations that led to the best accuracies (τ_*mem*_>420*ms* and τ_*syn*_ < 70*ms*) resulted in sparser activity, while combinations that led to the worst accuracies resulted in higher spiking activity. This is counter-intuitive, and there might be a sweet spot where a sufficient number of spikes leads to a an optimal accuracy. However, we were not able to see this sweet spot since we did not try to explicitly increase the sparsity until we see a decrease in accuracy if there are not enough spikes.

**Figure 4:**
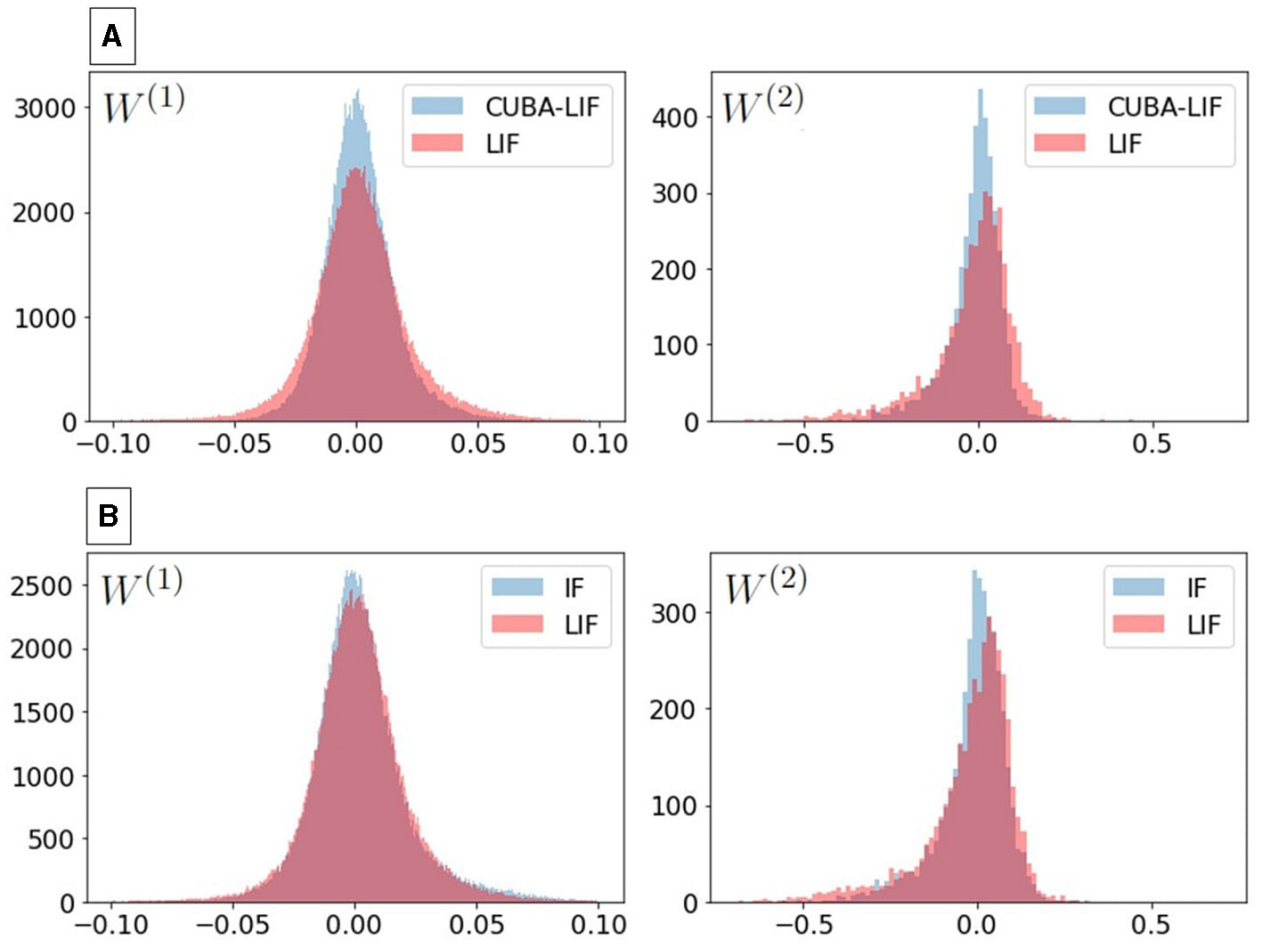
Trained weights distributions for: **(A)** LIF vs. CUBA-LIF for the weight matrices (left) *W*^(1)^ and (right) *W*^(2)^. The standard deviation in LIF (1.99 × 10^−2^ for *W*^(1)^ and 1.15 × 10^−1^ for *W*^(2)^) is higher than CUBA-LIF (1.44 × 10^−2^ for *W*^(1)^ and 0.77 × 10^−1^ for *W*^(2)^). **(B)** IF vs. LIF for the weight matrices (left) *W*^(1)^ and (right) *W*^(2)^. The standard deviation of *W*^(2)^ in LIF (12.58 × 10^−2^) is higher than IF (9.48 × 10^−2^).

Intuitively, we can assume that if all three neurons were to receive the same weighted sum of input, LIF neurons would produce comparatively sparser outputs due to their infinite synaptic leak and the layer-wise decay of spikes caused by its membrane leak that acts as a forgetting mechanism. For both datasets, we can see from Figure 3 that IF neurons produced slightly less spikes than LIF neurons in some experiments, which is counter-intuitive. In an attempt to understand the cause of this misleading intuition, we plotted the distributions of the trained weights for the LIF experiment that has the highest spiking activity (τ_*mem*_ = 420*ms*) to compare it with the weights distribution of the IF as depicted in Figure 4. Because it is hard to inspect the distributions visually, we calculated the mean and standard deviation. We can see that the standard deviation of the LIF's weight matrices *W*^(2)^ is higher than that of the IF. This result suggests that BPTT tailors the LIF model to increase the synaptic weights beyond what is needed for the IF model.

The CUBA-LIF model, on the other hand, was able achieve the sparsest activity among the three models for certain combinations of time constants despite its ability to sustain input spikes for longer duration. To that effect, we plotted the weights distributions of CUBA-LIF's experiment with τ_*mem*_ = 1, 680*ms* and τ_*syn*_ = 28*ms* and compared it with that of the LIF experiment that has the same τ_*mem*_ value. Again, we calculated the mean and standard deviation. We can see that the standard deviation of CUBA-LIF'S weight matrices *W*^(2)^ is higher than that of the LIF. Once more, this can be attributed to BPTT. Therefore, the results clearly indicate that the leakages do not necessarily lead to sparser activity.

### 3.3 Impact of explicit recurrences

To study the effect of recurrences on learning spatio-temporal patterns, we added explicit recurrent connections to neurons in the hidden layer and confronted the three neuron models in the context of a Recurrently-connected SNN (RSNN). Similar to the experiments in the FSNN, we performed a grid search across the same combinations of time constants for CUBA-LIF, a sweep for the same τ_*mem*_ values for LIF, and the same experiments for IF.

#### 3.3.1 Accuracy analysis in RSNN

**Figure 5:**
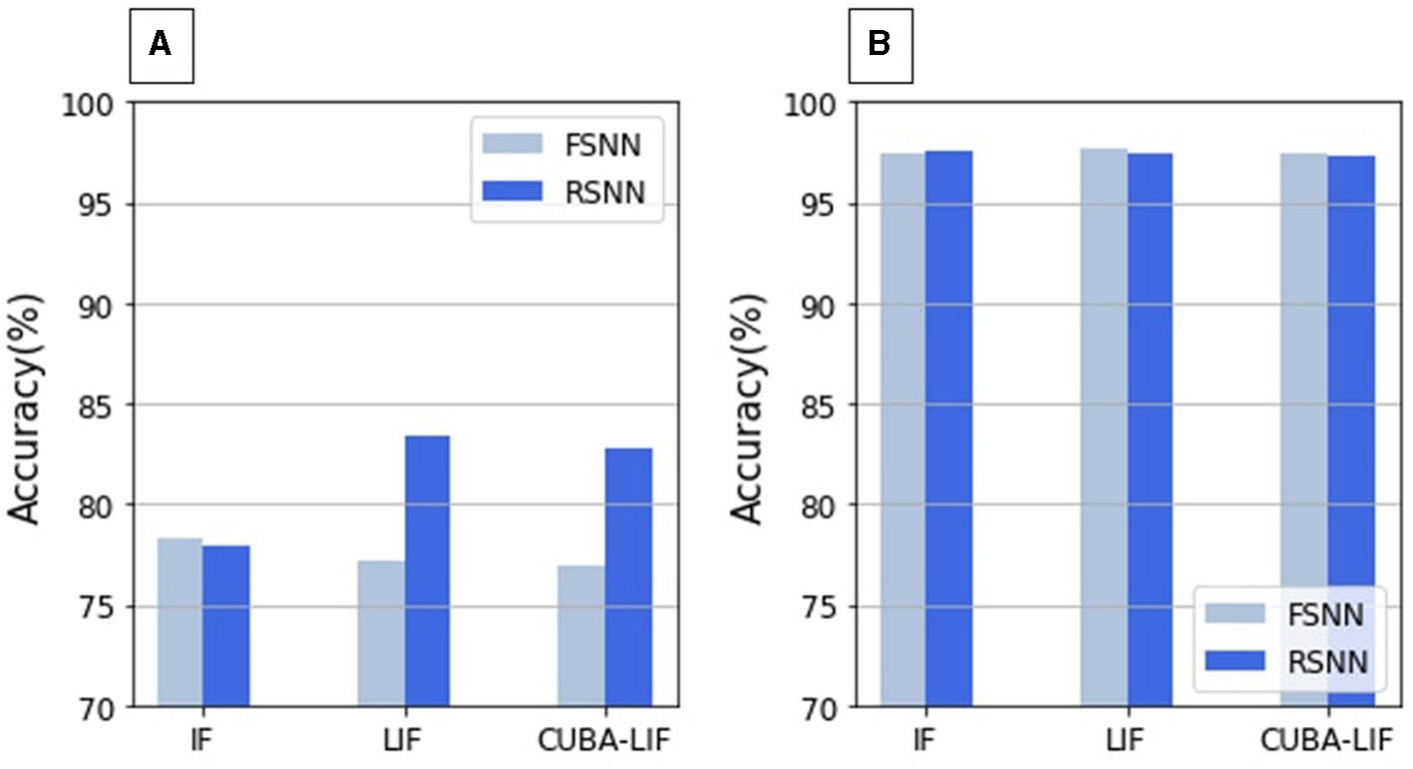
Best accuracies comparison between models in FSNN vs. RSNN. **(A)** SHD, **(B)** N-MNIST.

As shown in Table 3, results of the SHD dataset show that recurrent architectures reached a significantly higher performances than their feed-forward counterparts across all combinations of time constants for both CUBA-LIF and LIF. However, that is not the case for the N-MNIST. This is not surprising knowing the inherent ability of Recurrently Connected Neural Networks (RCNN) to handle time series and sequential data. For both datasets however, we can still observe the same trend as in FSNN such that τ_*mem*_ values below 420*ms* result in a significant decrease in accuracy for both CUBA-LIF and LIF, while CUBA-LIF performed better with smaller values of τ_*syn*_. Nevertheless, the LIF neuron reached the highest accuracy among the two. For the IF neuron, adding explicit recurrences reduced the accuracy by 0.43% on SHD and lead to comparable accuracy on N-MNIST. A comparison between the best accuracies obtained by the models in both FSNN and RSNN is presented if Figure 5.

**Table 3:** Three neuron models accuracy in RSNN.

	**LIF**	**CUBA-LIF**			
**(ms)**	τ_*syn*_ = 0 (α≈0) **(%)**	τ_*syn*_ = 14 (α≈0.368) **(%)**	τ_*syn*_ = 28 (α≈0.606) **(%)**	τ_*syn*_ = 70 (α≈0.818) **(%)**	τ_*syn*_ = 140 (α≈0.905) **(%)**
**A. SHD**					
τ_*mem*_ = 14 (β≈0.368)	44.67	58.56	73.54	75.26	73.19
τ_*mem*_ = 70 (β≈0.818)	70.51	76.41	79.64	79.41	74.59
τ_*mem*_ = 140 (β≈0.905)	78.34	80.48	81.25	78.64	75.96
τ_*mem*_ = 420 (β≈0.967)	82.72	81.96	81.71	77.05	75.15
τ_*mem*_ = 700 (β≈0.980)	83.06	82.44	80.65	78.81	75.91
τ_*mem*_ = 1120 (β≈0.987)	83.24	82.74	80.73	78.89	75.52
τ_*mem*_ = 1680 (β≈0.992)	83.41	82.25	80.68	79.83	76.06
τ_*mem*_ = ∞(β≈1)	77.93*				
**B. N-MNIST**					
τ_*mem*_ = 14 (β≈0.368)	96.18	97.22	97.14	96.83	96.92
τ_*mem*_ = 70 (β≈0.818)	97.10	97.27	97.09	96.78	96.29
τ_*mem*_ = 140 (β≈0.905)	97.28	97.24	97.00	96.62	96.11
τ_*mem*_ = 420 (β≈0.967)	97.39	97.26	97.18	96.27	95.57
τ_*mem*_ = 700 (β≈0.980)	97.44	97.35	96.81	96.08	95.88
τ_*mem*_ = 1120 (β≈0.987)	97.48	97.32	96.74	96.20	95.72
τ_*mem*_ = 1680 (β≈0.992)	97.41	97.22	96.80	96.21	95.95
τ_*mem*_ = ∞(β≈1)	97.54*				

^*^IF Neuron.

Given IF's good performance in FSNN with both datasets and inferior performance in RSNN with SHD, it becomes clear that leakages are important when there are both temporal information in the data and a recurrent topology in the network. This result is the most important finding of this work and our unique contribution to the neuromorphic computing literature. To the best of our knowledge, the highest accuracies we were able to reach on the SHD dataset are very close to state-of-the-art results (Dampfhoffer et al., 2022). Table 4 compares our best results with other works in the literature. In the study done by Cramer et al. (2022), the best accuracy that reached 79.9% corresponds to time constants combination of τ_*mem*_ = 80*ms* and τ_*syn*_ = 40*ms*. On the other hand, this work yielded compelling results, demonstrating that we could achieve accuracies on par with the results obtained by Cramer et al. (2022) as shown in Table 3, while also surpassing it when extending the time constants range beyond 420*ms* and further explore new values.

**Table 4:** Comparison of our results with related works.

	**Neuron model**	**Standard training (%)**	Heterogeneous training* (%)
Cramer et al. (2022)	**CUBA-LIF**	79.9	-
Perez-Nieves et al. (2021)	**CUBA-LIF**	71.7	82.7
Dampfhoffer et al. (2022)	**CUBA-LIF**	**83.7**	-
	**LIF**	80.6	-
Our work	**CUBA-LIF**	82.74	82.84
	**LIF**	**83.41**	**83.47**

^*^Homogeneous initialization. The bold values indicate the best accuracies that have been highlighted.

#### 3.3.2 Sparsity analysis in RSNN

Similar to what we did in FSNN, we recorded the spiking activity of neurons in the hidden layer when the test set is inferred. SHD spikes count recordings plotted in Figure 3A show that explicit recurrent connections increase activity in all neurons for every combination of time constants. On average, we have 53.55% increase in spiking activity for CUBA-LIF, 53.35% for LIF, and 53.58% for IF. N-MNIST spikes count, on the other hand, did not increase for every combination time constants. It even decreased for some as shown in Figure 3B. On average, we have 3.89% increase for CUBA-LIF, 16.78% for LIF, and 15.75% for IF.

It is hard to say whether or not the bigger increase in spiking activity for SHD contributed to its improved classification accuracy given that we saw similar increase for IF neurons but a worsened performance. Given the CUBA-LIF experiments that resulted in classification performance that is almost as good as that of the LIF also resulted in the slightest increase in spiking activity. The CUBA-LIF model could be more suitable for low power applications especially if tuned better to reach even higher accuracy.

Once again, we can observe that the time constant combinations leading to the highest accuracies (τ_*mem*_>420*ms* and τ_*syn*_ < 70*ms*) added the fewest number of spikes, whereas those leading to the worst accuracies (τ_*mem*_ < 420*ms* and τ_*syn*_>70*ms*) added the greatest number of spikes. This result is particularly noticeable in the case of the SHD dataset.

### 3.4 Impact of neural heterogeneity

Most existing learning methods learn the synaptic weights only while requiring a manual tuning of leakages-related parameters similar to our previously presented experiments. These parameters are chosen to be the same for all neurons, which could limit the diversity and expressiveness of SNNs. In biological brains, neuronal cells have different time constants with distinct stereotyped distributions depending on the cell type (Hawrylycz et al., 2012; Manis P. et al., 2019; Manis P. B. et al., 2019). To assess whether this heterogeneity plays an important functional role or is just a byproduct of noisy developmental processes, several studies (Fang, 2020; Perez-Nieves et al., 2021) incorporated learnable time constants in the training process and found an enhanced performance as result. In an attempt to reduce time constants tuning efforts, we adopted a similar approach by enabling the training of time constants. This allows us to determine if this training indeed improves performance and confirms their findings. Hence, τ_*mem*_ and τ_*syn*_ will not be treated as hyper-parameters, but learned parameters along with the synaptic weights. We refer to this training process as heterogeneous training. Since the IF neuron has fixed values of time constants: τ_*mem*_ = ∞ and τ_*syn*_ = 0, it is not concerned with heterogeneous training. On the other hand, LIF neuron has a fixed τ_*syn*_ equal to zero but a variable τ_*mem*_ which we were able to train. For CUBA-LIF, both time constants are trained.

To evaluate the performance of incorporating learnable time constants in comparison with the standard training in our previously presented experiments, we compare two different conditions: values of time constants could be either all initialized to a single value and then trained (homogeneous initialization), or initialized randomly according to a uniform distribution and then also trained (heterogeneous initialization). We also conducted these experiments in both FSNN and RSNN.

#### 3.4.1 Homogeneous initialization

First, we initialized τ_*mem*_ and τ_*syn*_ to the same values we used in our grid search for both CUBA-LIF and LIF and trained them along with the synaptic weights. We found that incorporating learnable time constants did not have a profound impact on both datasets. As can be seen in Table 5, the best accuracies obtained with heterogeneous training are slightly higher than that of the standard training for the SHD. Conversely, N-MNIST reached the best accuracies with standard training. the results also show that performance is still sensitive to initial tuning of time constants since we can observe the same trend in the impact of τ_*mem*_ and τ_*syn*_ on accuracy for both CUBA-LIF and LIF neurons. Please refer to Supplementary material for results obtained with more time constants initialization values.

**Table 5:** Comparison between best accuracies of standard vs. heterogeneous training.

	**Neuron**	**Standard**	**Heterogeneous training**	
	**model**	**training (%)**	**Homog. init. (%)**	**Random init. (%)**
**A. FSNN**				
**SHD**	**CUBA-LIF**	76.94	**78.69**	64.84
	**LIF**	77.20	**79.84**	68.89
**N-MNIST**	**CUBA-LIF**	**97.41**	97.24	96.45
	**LIF**	**97.64**	97.41	97.58
**B. RSNN**				
**SHD**	**CUBA-LIF**	82.74	**82.84**	75.60
	**LIF**	83.41	**83.47**	83.18
**N-MNIST**	**CUBA-LIF**	**97.35**	97.14	96.11
	**LIF**	97.48	97.38	**97.69**

The bold values indicate the best accuracies that have been highlighted.

#### 3.4.2 Random initialization

Because time constant tuning is a daunting task and is often one of the largest costs for developing these models, time constants are initialized randomly according to a uniform distribution. The results shown in Table 5 indicate that, within an FSNN, performance significantly lags behind that achieved through heterogeneous training with homogeneous initialization, as well as standard training for both CUBA-LIF and LIF. This is especially apparent with the SHD dataset. In an RSNN, however, SHD accuracies drastically improved for both CUBA-LIF and LIF. In fact, the LIF neuron achieved an 83.18% accuracy which is on-par with the results obtained through other training approaches. For the CUBA-LIF, although 75.60% is far from sufficient compared to accuracies obtained earlier, it is promising given the amount of tuning required to achieve the best results with standard training.

Intuitively, the CUBA-LIF should be able to perform better than the LIF neuron or at least reach a similar accuracy, given that LIF is a special case of CUBA-LIF. However, due to the large search space of CUBA-LIF, it is possible that it converges to a sub-optimal solution compared to LIF within the same number of epochs. Further investigations are required to assess the impact of the current compartment of the CUBA-LIF by using datasets with a more complex temporal structure or longer sequences. These results tell us that heterogeneity in time constants could further improve performance and reduces time constants tuning efforts for data with information content in their temporal dynamics.

## 4 Discussion

In the neuro-scientific literature, it has been reported that leakages in biological neurons exist in many contexts such as synaptic transmission in the visual cortex (Artun et al., 1998) and sodium ion channels (Snutch and Monteil, 2007; Ren, 2011). Many spiking neuron models imitate this leaky behavior through an exponential decay in the synaptic current and membrane potential. Other models prioritize computational efficiency by removing the leakage. To tackle the lack in understanding of the effect of these leakages from the modeling perspective, we confronted three spiking neuron models with variable degrees of leaky behavior, namely the CUBA-LIF, LIF, and IF, in classification tasks with a number of degrees of freedom.

We first trained SNNs using the three neuron models with a feed-forward network to classify visual patterns of written digits from the N-MNIST dataset and auditory information of spoken digits from the SHD datasets. Surprisingly, the IF model, despite the absence of leaky behavior and the resulting lack of inherent temporal dynamics, slightly outperformed the other models on the SHD by reaching an accuracy of 78.36 ± 0.87%, and closely matched the best of LIF model accuracy on the N-MNIST by reaching 97.50 ± 0.06%. CUBA-LIF on the other hand, had the inferior performance among the three models on both datasets despite its intrinsic temporal dynamics caused by both synaptic and membrane leaks. Both LIF and CUBA-LIF saw a drastic decrease in accuracy when τ_*mem*_ is less than 420*ms*, which leads to a fast decay in membrane potential and loss of information. We also found that CUBA-LIF reached its highest accuracies when its dynamics are close to those of the LIF. We conclude that leakages do not necessarily lead to improved performances even on temporally complex tasks when using feed-forward networks. In terms of sparsity, it is IF to see sparser activity in IF neurons and CUBA-LIF neurons with smaller values of τ_*syn*_ than their LIF counterpart. Upon inspection of the trained weights distributions, it seems that BPTT is tailoring LIF neurons to have bigger weights, and hence more spikes. Therefore, leakages do not always lead to sparser activity. Furthermore, we noticed that very low spiking activity resulted in the worst classification performance on the SHD. Very high spiking activity associated with bigger τ_*syn*_ values also resulted in a worsened performance. These results suggest that there is a sweet-spot where a sufficient amount of spikes produce an optimal classification accuracy.

Overall, IF neurons are sufficient when using data without temporal information or a network without recurrence in terms of classification accuracy and sparsity. It suggests that the fundamental ingredient of spiking neurons is their statefullness, i.e., having an internal state with an implicit recurrence, even without leakage. Furthermore, they offer a better alternative if we consider digital neuromorphic hardware design that is based on application-specific needs. IF neurons could be very cheap in terms of hardware resources, as they only perform additions for the input integration and a comparison for the output evaluation. In contrast, the LIF and CUBA-LIF neurons require multipliers to implement the leakage in their current and/or voltage compartments as shown in Table 6, thus resulting in more expensive hardware.

**Table 6:** Number of multiplication, addition, and comparison operations per spiking neuron at each time step, where N is the number of inputs (feedforward and/or recurrent) to the neuron and P is the percentage of those inputs that receive a spike.

**Neuron model**	**IF**	**LIF**	**CUBA-LIF**
Multiplications	0	1	2
Additions	*N*×*P*	*N*×*P*	*N*×*P*+1
Comparisons	1	1	1

Next, we added explicit recurrent connections to the neurons in the hidden layer. Expectedly, we saw a big improvement in accuracy for the SHD that has a rich temporal structure and no improvement at all for the N-MNIST that has mostly spatial structure. However, recurrences did not have any impact on the IF neuron on both datasets. Therefore, we conclude that the inherent temporal dynamics introduced by the leakages are only necessary when we use both data with a rich temporal structure and a neural network with a explicit recurrence. The best SHD accuracies we were able to obtained in a RSNN were very close to state-of-the-art results (Dampfhoffer et al., 2022) such that we reached 82.74 ± 0.17% with CUBA-LIF and 83.41 ± 0.37% with the LIF. In terms of sparsity, we saw a bigger increase in spiking activity with the SHD than the N-MNIST. In both datasets, the CUBA-LIF neurons with the best time constants combinations added the smallest number of spikes, which gives them an advantage in sparsity compared to LIF neurons.

Finally, we introduced heterogeneity in the considered spiking neurons by incorporating learnable time constants in the training process following two approaches: homogeneous initialization and random initialization. Heterogeneous training with homogeneous initialization slightly improved performance on the SHD, which has a complex temporal structure. The best SHD accuracies we obtained with heterogeneous training in RSNN were also very close to state-of-the-art results (Dampfhoffer et al., 2022) with 82.84 ± 1.17% for CUBA-LIF and 83.47 ± 2.12% for LIF. However, results are very sensitive to initial values of time constants. On the other hand, random initialization did not improve performance but proved it can be promising given the 83.18 ± 0.19% achieved by the LIF with the SHD. For the CUBA-LIF, however, further investigations are required to assess the impact of its current compartment.

## 5 Conclusion

In this work we explored the effect of spiking neurons synaptic and membrane leakages, network explicit recurrences and time constants heterogeneity on event-based spatio-temporal pattern recognition. The main findings of our work can be summarized as follows:

- Neural leakages are only necessary when there are both temporal information in the data and explicit recurrent connections in the network.
- Neural leakages do not necessarily lead to sparser spiking activity in the network.
- Time constants heterogeneity slightly improves performance and reduces time constants tuning efforts on data with a rich temporal structure and does not affect performance on data with a spatial structure.

This work supports the identification of the right level of model abstraction of biological evidences needed to build efficient application-specific neuromorphic hardware. This is a crucial analysis for advancing the field beyond state-of-the-art, especially when constrains on resources are critical (e.g., edge computing). In fact, when using digital neuromorphic architectures, IF neurons have been shown to be 2 × smaller and more power-efficient than formal Perceptrons (Khacef et al., 2018). It is nevertheless not clear how this gain evolves when adding a multiplier to implement a LIF or CUBA-LIF neuron. Further works will focus on implementing these two architectures in FPGAs for fast prototyping. In addition, IF neurons give the possibility to implement a digital asynchronous processing purely driven by the input, since there is no inherent temporal dynamics in the spiking neurons. On the other hand, LIF and CUBA-LIF neurons require algorithmic time-steps where the leakage is updated regardless of the presence of input spikes. Further works will explore the impact of both paradigms in energy-efficiency on the Loihi neuromorphic chip (Davies et al., 2018).

Furthermore, it is important to mention that our results only hold in benchmarking so far. In a real-world scenario such as continuous keyword spotting, there can be more noise in the data but also in void. Hence, when using the IF neurons that do not have any leakage, this noise can accumulate and create false positives and degrade the performance. Indeed, the low-pass filtering effect of the spiking neurons leakages has been shown to eliminate high frequency components from the input and enhance the noise robustness of SNNs, especially in real-world environments (Chowdhury et al., 2021). In addition, given that the LIF model achieved a superior performance when compared to the CUBA-LIF, it is important to investigate where the latter could perform better. More complex tasks could show such a gain for the CUBA-LIF neuron, because of its current compartment which is an extra state that gives more potential for spatio-temporal feature extraction. Finally, spiking neural networks in neuromorphic hardware can be used beyond fast and efficient inference, by adding adaptation through local synaptic plasticity (Qiao et al., 2015; Khacef et al., 2022; Quintana et al., 2022). In this context, the impact of the leakage can be different, as the inherent temporal dynamics is required in some plasticity mechanisms (Brader et al., 2007; Clopath et al., 2010) for online learning.

## Data availability statement

The original contributions presented in the study are included in the article/Supplementary material, further inquiries can be directed to the corresponding author.

## Author contributions

LK and EC contributed to the conception and design of the study. MB worked on the software implementation and run the experiments. LK and DC supervised the work. All authors contributed to the manuscript writing and approved the submitted version.

## References

[B1] Abderrahmane N. Miramond B. Kervennic E. Girard A. (2022). “Spleat: spiking low-power event-based architecture for in-orbit processing of satellite imagery,” in 2022 International Joint Conference on Neural Networks (IJCNN) (Padua: IEEE WCCI Congress), 1–10.

[B2] Amir A. Taba B. Berg D. Melano T. McKinstry J. Di Nolfo C.. (2017). “A low power, fully event-based gesture recognition system,” in 2017 IEEE Conference on Computer Vision and Pattern Recognition (CVPR) (Honolulu, HI), 7388–7397.

[B3] Artun Ö. B. Shouval H. Z. Cooper L. N. (1998). The effect of dynamic synapses on spatiotemporal receptive fields in visual cortex. Proc. Natl. Acad. Sci. U.S.A. 95, 11999–12003. 9751779 10.1073/pnas.95.20.11999 PMC21754

[B4] Bouvier M. Valentian A. Mesquida T. Rummens F. Reyboz M. Vianello E.. (2019). Spiking neural networks hardware implementations and challenges: a survey. J. Emerg. Technol. Comput. Syst. 15, 1–35. 10.1145/3304103

[B5] Brader J. Senn W. Fusi S. (2007). Learning real world stimuli in a neural network with spike-driven synaptic dynamics. Neural Comput. 19, 2881–2912. 10.1162/neco.2007.19.11.2881 17883345

[B6] Ceolini E. Frenkel C. Shrestha S. B. Taverni G. Khacef L. Payvand M.. (2020). Hand-gesture recognition based on EMG and event-based camera sensor fusion: a benchmark in neuromorphic computing. Front. Neurosci. 14, 637. 10.3389/fnins.2020.00637 32903824 PMC7438887

[B7] Chicca E. Stefanini F. Bartolozzi C. Indiveri G. (2014). Neuromorphic electronic circuits for building autonomous cognitive systems. Proc. IEEE 102, 1367–1388. 10.1109/JPROC.2014.2313954

[B8] Chowdhury S. S. Lee C. Roy K. (2021). Towards understanding the effect of leak in spiking neural networks. Neurocomputing 464, 83–94. 10.1016/j.neucom.2021.07.091

[B9] Clopath C. Büsing L. Vasilaki E. Gerstner W. (2010). Connectivity reflects coding: a model of voltage-based STDP with homeostasis. Nat. Neurosci. 13, 344–352. 10.1038/nn.2479 20098420

[B10] Cramer B. Stradmann Y. Schemmel J. Zenke F. (2022). The Heidelberg spiking data sets for the systematic evaluation of spiking neural networks. IEEE Trans. Neural Netw. Learn. Syst. 33, 2744–2757. 10.1109/TNNLS.2020.3044364 33378266

[B11] Dampfhoffer M. Mesquida T. Valentian A. Anghel L. (2022). “Investigating current-based and gating approaches for accurate and energy-efficient spiking recurrent neural networks,” in Artificial Neural Networks and Machine Learning – ICANN 2022, eds Pimenidis E. Angelov P. Jayne C. Papaleonidas A. Aydin M. (Cham: Springer Nature), 359–370.

[B12] Davies M. Srinivasa N. Lin T.-H. Chinya G. Cao Y. Choday S. H.. (2018). Loihi: a neuromorphic manycore processor with on-chip learning. IEEE Micro 38, 82–99. 10.1109/MM.2018.112130359

[B13] Davies M. Wild A. Orchard G. Sandamirskaya Y. Guerra G. A. F. Joshi P.. (2021). Advancing neuromorphic computing with loihi: a survey of results and outlook. Proc. IEEE 109, 911–934. 10.1109/JPROC.2021.3067593

[B14] Eliasmith C. (2013). How to Build a Brain: A Neural Architecture for Biological Cognition. Oxford University Press. 10.1093/acprof:oso/9780199794546.001.0001

[B15] Fang W. (2020). Incorporating learnable membrane time constant to enhance learning of spiking neural networks. arXiv preprint arXiv:2007.05785. 10.48550/arXiv.2007.05785

[B16] Frenkel C. Legat J.-D. Bol D. (2019). Morphic: a 65-nm 738k-synapse/mm^2^ quad-core binary-weight digital neuromorphic processor with stochastic spike-driven online learning. IEEE Trans. Biomed. Circ. Syst. 13, 999–1010. 10.1109/TBCAS.2019.2928793 31329562

[B17] Gallego G. Delbruck T. Orchard G. Bartolozzi C. Taba B. Censi A.. (2022). Event-based vision: a survey. IEEE Trans. Pattern Anal. Mach. Intell. 44, 154–180. 10.1109/TPAMI.2020.3008413 32750812

[B18] Gerstner W. Kistler W. M. Naud R. Paninski L. (2014). Neuronal Dynamics: From Single Neurons to Networks and Models of Cognition. Cambridge University Press. 10.1017/CBO9781107447615

[B19] Goodfellow I. Bengio Y. Courville A. (2016). Deep Learning. MIT Press.

[B20] Hawrylycz M. Lein E. Guillozet-Bongaarts A. Shen E. Ng L. Miller J.. (2012). An anatomically comprehensive atlas of the adult human brain transcriptome. Nature 489, 391–399. 10.1038/nature11405 22996553 PMC4243026

[B21] Hodgkin A. L. Huxley A. F. (1952). A quantitative description of membrane current and its application to conduction and excitation in nerve. J. Physiol. 117, 500–544. 12991237 10.1113/jphysiol.1952.sp004764 PMC1392413

[B22] Indiveri G. Linares-Barranco B. Hamilton T. van Schaik A. Etienne-Cummings R. Delbruck T.. (2011). Neuromorphic silicon neuron circuits. Front. Neurosci. 5, 73. 10.3389/fnins.2011.00073 21747754 PMC3130465

[B23] Iyer L. R. Chua Y. Li H. (2021). Is neuromorphic mnist neuromorphic? Analyzing the discriminative power of neuromorphic datasets in the time domain. Front. Neurosci. 15, 608567. 10.3389/fnins.2021.608567 33841072 PMC8027306

[B24] Izhikevich E. (2003). Simple model of spiking neurons. IEEE Trans. Neural Netw. 14, 1569–1572. 10.1109/TNN.2003.820440 18244602

[B25] Izhikevich E. (2004). Which model to use for cortical spiking neurons? IEEE Trans. Neural Netw. 15, 1063–1070. 10.1109/TNN.2004.832719 15484883

[B26] Khacef L. Abderrahmane N. Miramond B. (2018). “Confronting machine-learning with neuroscience for neuromorphic architectures design,” in 2018 International Joint Conference on Neural Networks (IJCNN) (Rio de Janeiro), 1–8. 10.1109/IJCNN.2018.8489241

[B27] Khacef L. Klein P. Cartiglia M. Rubino A. Indiveri G. Chicca E. (2022). Spike-based local synaptic plasticity: a survey of computational models and neuromorphic circuits. arXiv preprint arXiv:2209.15536. 10.48550/arXiv.2209.15536

[B28] Kingma D. P. Ba J. (2014). Adam: a method for stochastic optimization. arXiv preprint arXiv:1412.6980. 10.48550/arXiv.1412.6980

[B29] Kistler W. M. Gerstner W. Hemmen J. L. v. (1997). Reduction of the Hodgkin-Huxley equations to a single-variable threshold model. Neural Comput. 9, 1015–1045.

[B30] Liu Q. Richter O. Nielsen C. Sheik S. Indiveri G. Qiao N. (2019). “Live demonstration: face recognition on an ultra-low power event-driven convolutional neural network asic,” in 2019 IEEE/CVF Conference on Computer Vision and Pattern Recognition Workshops (CVPRW) (Long Beach, CA), 1680–1681. 10.1109/CVPRW.2019.00213

[B31] Liu S.-C. van Schaik A. Minch B. A. Delbruck T. (2010). “Event-based 64-channel binaural silicon cochlea with q enhancement mechanisms,” in 2010 IEEE International Symposium on Circuits and Systems (ISCAS) (Paris), 2027–2030. 10.1109/ISCAS.2010.5537164

[B32] Maass W. (1997). Networks of spiking neurons: the third generation of neural network models. Neural Netw. 10, 1659–1671.

[B33] Maass W. Natschloger T. Markram H. (2002). Real-time computing without stable states: a new framework for neural computation based on perturbations. Neural Comput. 14, 2531–2560. 10.1162/089976602760407955 12433288

[B34] Manis P. Kasten M. R. Ruili Xie (2019). Raw voltage and current traces for current-voltage (IV) relationships for cochlear nucleus neurons. Figshare. Dataset. 10.6084/m9.figshare.8854352.v1

[B35] Manis P. B. Kasten M. R. Xie R. (2019). Classification of neurons in the adult mouse cochlear nucleus: linear discriminant analysis. bioRxiv. 10.1101/594713 31581200 PMC6776397

[B36] Mead C. Conway L. (1980). Introduction to VLSI Systems. Reading, MA: Addison-Wesley.

[B37] Merolla P. A. Arthur J. V. Alvarez-Icaza R. Cassidy A. S. Sawada J. Akopyan F.. (2014). A million spiking-neuron integrated circuit with a scalable communication network and interface. Science 345, 668–673. 10.1126/science.1254642 25104385

[B38] Muller-Cleve S. F. Fra V. Khacef L. Pequeao-Zurro A. Klepatsch D. Forno E.. (2022). Braille letter reading: a benchmark for spatio-temporal pattern recognition on neuromorphic hardware. Front. Neurosci. 16, 951164. 10.3389/fnins.2022.951164 36440280 PMC9695069

[B39] Neftci E. O. Mostafa H. Zenke F. (2019). Surrogate gradient learning in spiking neural networks: bringing the power of gradient-based optimization to spiking neural networks. IEEE Signal Process. Mag. 36, 51–63. 10.1109/MSP.2019.2931595

[B40] Orchard G. Jayawant A. Cohen G. K. Thakor N. (2015). Converting static image datasets to spiking neuromorphic datasets using saccades. Front. Neurosci. 9, 437. 10.3389/fnins.2015.00437 26635513 PMC4644806

[B41] Perez-Nieves N. Leung V. C. Dragotti P. L. Goodman D. F. (2021). Neural heterogeneity promotes robust learning. Nat. Commun. 12, 1–9. 10.1038/s41467-021-26022-3 34608134 PMC8490404

[B42] Qiao N. Mostafa H. Corradi F. Osswald M. Stefanini F. Sumislawska D.. (2015). A reconfigurable on-line learning spiking neuromorphic processor comprising 256 neurons and 128k synapses. Front. Neurosci. 9, 141. 10.3389/fnins.2015.00141 25972778 PMC4413675

[B43] Quintana F. M. Perez-Peña F. Galindo P. L. (2022). Bio-plausible digital implementation of a reward modulated STDP synapse. Neural Comput. Appl. 34, 15649–15660. 10.1007/s00521-022-07220-6

[B44] Rabaey J. M. Verhelst M. Boeck J. D. Enz C. Greve K. D. Ionescu A. M.. (2019). AI at the Edge - a Roadmap. Technical report by IMEC, KU Leuven, Ghent University, VUB, EPFL, ETH Zurich and UC Berkeley.

[B45] Ren D. (2011). Sodium leak channels in neuronal excitability and rhythmic behaviors. Neuron 72, 899–911. 10.1016/j.neuron.2011.12.007 22196327 PMC3247702

[B46] Schuman C. D. Potok T. E. Patton R. M. Birdwell J. D. Dean M. E. Rose G. S.. (2017). A survey of neuromorphic computing and neural networks in hardware. arXiv preprint arXiv:1705.06963. 10.48550/arXiv.1705.06963

[B47] Shalf J. (2020). The future of computing beyond Moore's law. Philos. Trans. R. Soc. A Math. Phys. Eng. Sci. 378, 20190061. 10.1098/rsta.2019.0061 31955683

[B48] Snutch T. P. Monteil A. (2007). The sodium “leak” has finally been plugged. Neuron 54, 505–507. 10.1016/j.neuron.2007.05.005 17521564

[B49] Thompson N. C. Greenewald K. Lee K. Manso G. F. (2021). Deep learning's diminishing returns: the cost of improvement is becoming unsustainable. IEEE Spectrum 58, 50–55. 10.1109/MSPEC.2021.9563954

[B50] Thompson N. C. Greenewald K. H. Lee K. Manso G. F. (2020). The computational limits of deep learning. arXiv preprint arXiv:2007.05558. 10.48550/arXiv.2007.05558

