# Supplementary material for: Impact of spiking neurons leakages and network recurrences on event-based spatio-temporal pattern recognition
Source: Front Neurosci. 2023 Nov 24;17:1244675. doi: 10.3389/fnins.2023.1244675 (PMC10704147; doi:10.3389/fnins.2023.1244675)
Supplement: Supplementary file 1 [file Table_1.PDF]

Supplementary Material

Table S1 and Table S2 show the grid search results obtained by initializing time constants to the same values of standard training and incorporating them in the training process. the results show that performance is still sensitive to initial tuning of time constants.

Table S1. Heterogeneous training accuracy in FSNN for different initial values of time constants.

Table 1a. SHD

	LIF	CUBA-LIF			
**(ms)**	$\tau_{syn} = 0$ ($\alpha \approx 0$)	$\tau_{syn} = 14$ ($\alpha \approx 0.368$)	$\tau_{syn} = 28$ ($\alpha \approx 0.606$)	$\tau_{syn} = 70$ ($\alpha \approx 0.818$)	$\tau_{syn} = 140$ ($\alpha \approx 0.905$)
$\tau_{mem} = 420$ ($\beta \approx 0.967$)	75.67%	76.07%	74.80%	71.52%	66.23%
$\tau_{mem} = 700$ ($\beta \approx 0.980$)	78.09%	76.78%	75.44%	72.62%	67.40%
$\tau_{mem} = 1120$ ($\beta \approx 0.987$)	78.02%	76.23%	75.52%	71.94%	67.89%
$\tau_{mem} = 1680$ ($\beta \approx 0.992$)	79.84%	78.69%	76.78%	72.79%	66.79%

Table 1b. N-MNIST

	LIF	CUBA-LIF			
**(ms)**	$\tau_{syn} = 0$ ($\alpha \approx 0$)	$\tau_{syn} = 14$ ($\alpha \approx 0.368$)	$\tau_{syn} = 28$ ($\alpha \approx 0.606$)	$\tau_{syn} = 70$ ($\alpha \approx 0.818$)	$\tau_{syn} = 140$ ($\alpha \approx 0.905$)
$\tau_{mem} = 420$ ($\beta \approx 0.967$)	97.41%	97.08%	96.75%	95.86%	95.68%
$\tau_{mem} = 700$ ($\beta \approx 0.980$)	97.24%	97.24%	96.74%	95.91%	95.65%
$\tau_{mem} = 1120$ ($\beta \approx 0.987$)	97.37%	97.15%	96.65%	96.14%	95.51%
$\tau_{mem} = 1680$ ($\beta \approx 0.992$)	97.34%	97.08%	96.86%	96.20%	95.50%

Table S2. Heterogeneous training accuracy in RSNN for different initial values of time constants.

Table 2a. SHD

	LIF	CUBA-LIF			
**(ms)**	$\tau_{syn} = 0$ ($\alpha \approx 0$)	$\tau_{syn} = 14$ ($\alpha \approx 0.368$)	$\tau_{syn} = 28$ ($\alpha \approx 0.606$)	$\tau_{syn} = 70$ ($\alpha \approx 0.818$)	$\tau_{syn} = 140$ ($\alpha \approx 0.905$)
$\tau_{mem} = 420$ ($\beta \approx 0.967$)	83.27%	81.48%	81.48%	79.00%	75.58%
$\tau_{mem} = 700$ ($\beta \approx 0.980$)	83.47%	82.84%	81.75%	76.90%	75.52%
$\tau_{mem} = 1120$ ($\beta \approx 0.987$)	83.10%	82.21%	81.07%	78.05%	77.04%
$\tau_{mem} = 1680$ ($\beta \approx 0.992$)	83.10%	82.35%	80.90%	79.29%	76.47%

Table 2b. N-MNIST

	LIF	CUBA-LIF			
**(ms)**	$\tau_{syn} = 0$ ($\alpha \approx 0$)	$\tau_{syn} = 14$ ($\alpha \approx 0.368$)	$\tau_{syn} = 28$ ($\alpha \approx 0.606$)	$\tau_{syn} = 70$ ($\alpha \approx 0.818$)	$\tau_{syn} = 140$ ($\alpha \approx 0.905$)
$\tau_{mem} = 420$ ($\beta \approx 0.967$)	97.25%	97.07%	96.74%	96.08%	95.64%
$\tau_{mem} = 700$ ($\beta \approx 0.980$)	97.24%	96.92%	96.79%	96.17%	95.59%
$\tau_{mem} = 1120$ ($\beta \approx 0.987$)	97.38%	97.14%	96.55%	96.00%	95.76%
$\tau_{mem} = 1680$ ($\beta \approx 0.992$)	97.23%	97.02%	96.49%	96.00%	95.41%
